# Pressor responsiveness to angiotensin II in female mice is enhanced with age: role of the angiotensin type 2 receptor

**DOI:** 10.1186/s13293-014-0013-7

**Published:** 2014-09-16

**Authors:** Katrina M Mirabito, Lucinda M Hilliard, Geoffrey A Head, Robert E Widdop, Kate M Denton

**Affiliations:** 1 Department of Physiology, Monash University, Building 13F, Victoria 3800, Australia; 2 Department of Pharmacology, Monash University, Building 13E, Victoria 3800, Australia; 3 Baker IDI Heart and Diabetes Institute, Melbourne 3004, Victoria, Australia

**Keywords:** Angiotensin type 2 receptor, Hypertension, Aging, Renin angiotensin system, Females

## Abstract

**Background:**

The pressor response to angiotensin II (AngII) is attenuated in adult females as compared to males via an angiotensin type 2 receptor (AT_2_R)-dependent pathway. We hypothesized that adult female mice are protected against AngII-induced hypertension via an enhanced AT_2_R-mediated pathway and that in reproductively senescent females this pathway is no longer operative.

**Methods:**

Mean arterial pressure was measured via telemetry in 4-month-old (adult) and 16-month-old (aged) and aged ovariectomized (aged-OVX) wild-type and AT_2_R knockout (AT_2_R-KO) female mice during baseline and 14-day infusion of vehicle (saline) or AngII (600 ng/kg/min s.c.). Real-time reverse transcription polymerase chain reaction (RT-PCR) was used to determine renal gene expression of angiotensin receptors and angiotensin-converting enzyme 2 in response to 14-day treatment with vehicle or AngII.

**Results:**

Basal mean arterial pressure was similar between the groups. The pressor response to AngII was augmented in adult AT_2_R-KO compared to adult wild-type mice (29 ± 3 mmHg versus 10 ± 4 mmHg, respectively, on day 14 as compared to basal mean arterial pressure, *P* = 0.002). In wild-type mice, pressor responsiveness to AngII was augmented with age, such that the pressor response to AngII was similar between aged AT_2_R-KO and wild-type female mice (31 ± 4 mmHg versus 34 ± 3 mmHg, respectively, on day 14, *P* = 0.9). There were no significant differences in pressor responsiveness to AngII between aged and aged-OVX mice. Vehicle-treated aged wild-type mice had a lower renal AT_2_R/AT_1_R balance as compared to adult counterparts. In response to AngII, the renal AT_2_R/AT_1_R balance in aged wild-type females was greater than that observed in vehicle-treated aged wild-type females and adult wild-type females, yet the protective effects of AT_2_R activation were not restored.

**Conclusions:**

The protective role of the AT_2_R depressor pathway is lost with age in female mice. Therefore, targeting deficits in AT_2_R expression and/or signaling may represent a novel anti-hypertensive approach in aged females.

## 1 Background

Women of reproductive age are protected from hypertension and cardiovascular disease (CVD) relative to postmenopausal women [1]. In the years following menopause, arterial pressure increases, suggesting that the mechanisms that regulate arterial pressure are altered with age. Sex hormones are known to differentially regulate the renin angiotensin system (RAS), a key regulator of arterial pressure [2]. Previous studies have demonstrated that estrogen enhances the RAS depressor pathways, which encompasses the angiotensin-converting enzyme 2 (ACE2)/angiotensin (1-7) (Ang(1-7))/MasR pathway and the angiotensin type 2 receptor (AT_2_R) [3]-[5]. Thus, the RAS depressor pathways are thought to play a protective role in the setting of arterial pressure in adult females of reproductive age [6],[7].

Chronic angiotensin II (AngII)-induced hypertension is attenuated in female as compared to male rodents [4],[8],[9]. We have previously demonstrated that this sex difference in pressor responsiveness to AngII is associated with higher renal AT_2_R expression in females as compared to males [3],[4]. Moreover, AT_2_R deficiency increases the pressor responsiveness to AngII in adult female mice to a similar extent to that observed in age-matched male mice [4]. Collectively, these data indicate that the AT_2_R plays a central role in the attenuated pressor response to AngII-induced hypertension in females. In healthy adults, sexual dimorphism in the renal vascular response to AngII has been identified, and it has been suggested that the AT_2_R may be responsible for these differences [10],[11]. Given that AT_2_R expression is estrogen dependent [3]-[5], it is plausible that reproductive senescence may lead to lower AT_2_R expression and, in turn, increased pressor responsiveness to AngII in aged females.

In the present study, our aims were 2-fold: firstly, to determine if renal AT_2_R expression is reduced in aged reproductively senescent female mice and, secondly, to determine if the attenuated pressor response to AngII observed in adult female mice is still operative in aged reproductively senescent female mice. We hypothesized that adult female mice are protected against AngII-induced hypertension via an enhanced AT_2_R-mediated pathway and that with age and reproductive senescence this pathway is no longer operative.

## 2 Methods

Experiments were conducted in accordance with the Australian Code of Practice for the Care and Use of Animals for Scientific Purposes and approved by the Monash University School of Biomedical Sciences Animal Ethics Committee. Female FVB/N wild-type (WT) and AT_2_R knockout (AT_2_R-KO) mice, initially established by Hein et al. [12], were obtained at 12 weeks of age (Monash Animal Services). Animals were housed in an experimental room with temperature maintained at 24°C–26°C and a 12-h light-dark cycle. Mice had ad libitum access to normal salt diet (0.26% (*w*/*w*) NaCl; AIN93M, Specialty Feeds, Australia) and water.

### 2.1 Animal model

Mice were divided into 12 groups: adult (4-month-old) WT and AT_2_R-KO, aged (16-month-old) WT and AT_2_R-KO, and aged ovariectomized (OVX) WT and aged-OVX AT_2_R-KO receiving either vehicle or AngII for 14 days. OVX was performed at 14 months of age, as described previously [13]. Vaginal smears confirmed that the aged mice were in persistent estrus before commencement of the study.

### 2.2 Experimental protocol

Mice were anesthetized (2.2%–2.6% isoflurane in 40% O_2_-60% N_2_, Rhodia, Australia) for implantation of a radiotelemetry probe (TA11PA-C10, Data Sciences International, MN, USA) into the left carotid artery, as described previously [14]. Following a 10-day recovery period, basal mean arterial pressure (MAP), heart rate (HR), and locomotor activity were measured by sampling for 10 s every 10 min using Dataquest ART data acquisition system (Data Sciences International, MN, USA) for 3 days. Thereafter, mice were briefly anesthetized (as described above), for implantation of an osmotic pump into the midscapular region (ALZET model 1002, Durect Corp., Cupertino, CA) delivering AngII (600 ng/kg/min s.c.) or vehicle (0.9% saline 0.25 μl/h s.c.). MAP, HR, and locomotor activity were measured, as described above, over the 14-day treatment period. At the conclusion of the experiment, animals were anesthetized and killed by exsanguination via cardiac puncture.

### 2.3 Renal AT_1a_R, AT_1b_R, AT_2_R, ACE2, and MasR gene expression

RNA was extracted from kidneys following a 14-day infusion of vehicle or AngII using the RNeasy Mini Kit (Qiagen, Doncaster, Victoria, Australia). AT_1a_R, AT_1b_R, AT_2_R, MasR, and ACE2 gene expressions were analyzed by real-time quantitative reverse transcription polymerase chain reaction (RT-PCR) Realplex software with the Applied Biosystems 7900HT Fast RT-PCR system (Applied Biosystems, Life Technologies, Australia). Samples were run in triplicate using TaqMan gene expression assays (Applied Biosystems, Life Technologies, Australia) and 18S rRNA as the internal housekeeping gene. Reactions were set up on a 384-well PCR plate using an automated liquid handler (CAS-1200 liquid handler, Qiagen, Australia) as per the manufacturer's instructions (Applied Biosystems, Life Technologies, Australia). Calculations of relative expression were carried out using the comparative cycle of threshold fluorescence (2^−ΔΔCT^) method.

### 2.4 Statistical analysis

Data are presented as mean ± SEM. MAP, HR, and locomotor activity over the 14-day treatment period were analyzed using repeated measures analysis of variance (ANOVA) with the factors genotype (*P*_g_) and time (*P*_t_). All other data were analyzed using a one-way ANOVA followed by post hoc *t*-tests with Holm-Sidak correction to reduce the risk of type 1 error associated with multiple comparisons. Comparisons were made to evaluate the effect of age, genotype, or OVX. *P* ≤ 0.05 was considered statistically significant.

## 3 Results

### 3.1 Basal hemodynamics, locomotor activity, and body weight

Basal values were pooled for the adult, aged, and aged-OVX groups prior to vehicle or AngII infusion. Within groups, there were no differences in basal hemodynamics, locomotor activity or body weight between the animals that were allocated to vehicle or AngII treatment. Basal 24-h MAP was 93 ± 1 mmHg in adult WT and 94 ± 1 mmHg in adult AT_2_R-KO females (Table 1). Age did not increase basal MAP (Table 1). Moreover, OVX had no effect on basal MAP in aged females (Table 1). Basal HR was not different between the groups (Table 1). While locomotor activity was similar between the genotypes, there was a significant reduction in locomotor activity in the aged-OVX WT versus aged WT females (Table 1, *P* = 0.03). Aged female mice were 4–6 g heavier than their adult counterparts; however, this difference was not statistically significant (Table 1). There was no difference in body weight between aged and aged-OVX mice (Table 1).

**Table 1:** **Basal hemodynamics, activity, and body weight of adult, aged, and aged-OVX WT and AT**_**2**_**R-KO female mice**

	**MAP (mmHg)**	**HR (bpm)**	**Activity (units)**	**BW (g)**
Adult WT (*n* = 13)	93 ± 1	570 ± 16	6 ± 1	22 ± 2
Aged WT (*n* = 11)	94 ± 1	555 ± 13	7 ± 1	28 ± 2
Aged-OVX WT (*n* = 12)	91 ± 1	548 ± 20	4 ± 1*	31 ± 3
Adult AT_2_R-KO (*n* = 12)	94 ± 1	565 ± 12	8 ± 1	23 ± 1
Aged AT_2_R-KO (*n* = 14)	93 ± 1	594 ± 11	7 ± 1	27 ± 2
Aged-OVX AT_2_R-KO (*n* = 12)	91 ± 1	588 ± 13	5 ± 1	27 ± 3

Data are presented as 24 h mean ± SEM. Data were analyzed using a one-way ANOVA followed by selected post hoc *t*-tests with Holm-Sidak correction to reduce the risk of type 1 error associated with multiple comparisons. *MAP* mean arterial pressure, *HR* heart rate, *bpm* beats per minute, *BW* body weight, *g* grams. *P < 0.05 versus aged WT.

### 3.2 Hemodynamic and locomotor activity responses to treatment

**Figure 1:**
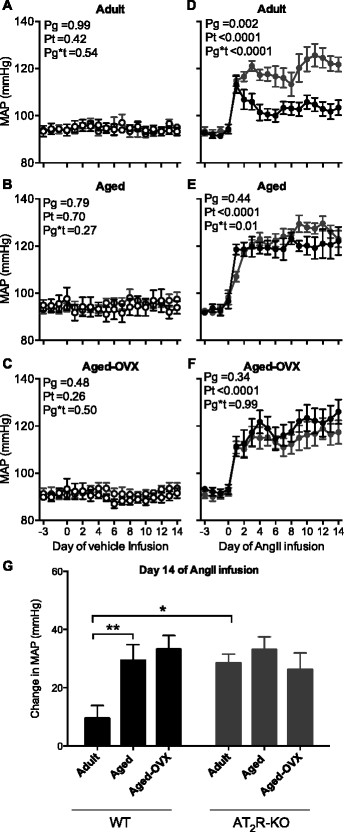
**Mean arterial pressure response to angiotensin II infusion.** Mean arterial pressure (MAP) during baseline and 14-day infusion of vehicle (0.9% saline, *n* = 4–8 per group; open symbols) in **(A)** adult, **(B)** aged, and **(C)** aged-OVX or AngII (600 ng/kg/min, *n* = 6–8 per group; closed symbols) in **(D)** adult, **(E)** aged, and **(F)** aged-OVX WT (black symbols) and AT_2_R-KO (grey symbols) female mice. **(G)** The change in MAP from baseline at day 14 of AngII infusion in WT (black symbols) and AT_2_R-KO (grey symbols) female mice. Data are presented as 24 h mean ± SEM. Data in (A–F) were analyzed using repeated measures ANOVA. The change in MAP on day 14 of AngII infusion was analyzed using a one-way ANOVA followed by post hoc *t*-tests with Holm-Sidak correction to reduce the risk of type 1 error associated with multiple comparisons. *P < 0.05; **P < 0.01.

There was no change in MAP in response to vehicle infusion in any group (Figure 1A–C). The pressor response to AngII was augmented in adult AT_2_R-KO versus adult WT mice (*P*_g_ = 0.002, Figure 1D). In contrast, the pressor response to AngII was similar between the aged WT and AT_2_R-KO mice (*P*_g_ = 0.9, Figure 1E). The MAP response to AngII over the 14-day treatment period was similar between the aged-OVX mice (*P*_g_ = 0.9, Figure 1F). Comparison of the HR and locomotor responses to treatment did not detect any differences between the groups (data not shown).

Pressor responsiveness on day 14 of AngII infusion was analyzed further to compare responses between each group. The increase in MAP, as compared to baseline, in response to AngII infusion was 10 ± 4 mmHg in adult WT females and 29 ± 3 mmHg in adult AT_2_R-KO females (*P* = V 0.02, Figure 1G). Between WT females, pressor responsiveness to AngII was enhanced in aged WT female as compared to adult WT female mice (*P* = 0.003, Figure 1G). The increase in MAP in response to AngII observed in the aged WT was comparable to that observed in adult AT_2_R-KO and aged AT_2_R-KO female mice (34 ± 3 mmHg, 29 ± 3 mmHg, and 31 ± 4 mmHg, respectively, Figure 1G). Moreover, there was no difference in pressor responsiveness to AngII between the aged and aged-OVX groups (Figure 1G).

### 3.3 Renal AT_1a_R, AT_1b_R, AT_2_R, MasR, and ACE2 gene expression

**Figure 2:**
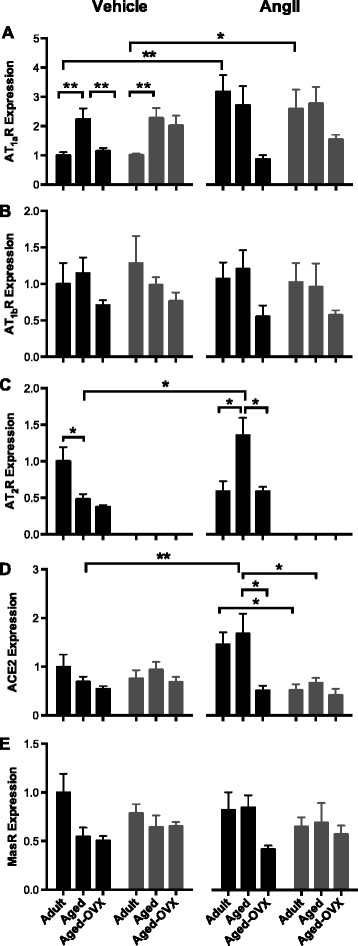
**Relative renal gene expression.** The relative renal gene expression of **(A)** AT_1a_R, **(B)** AT_1b_R, **(C)** AT_2_R, **(D)** ACE2, and **(E)** MasR in WT (black bars) and AT_2_R-KO (grey bars) adult, aged, and aged-OVX female mice following 14-day infusion of vehicle (0.9% saline s.c.) or AngII (600 ng/kg/min s.c.). Data are presented as mean ± SEM and are expressed relative to the adult WT vehicle-treated group. Data were analyzed using a one-way ANOVA followed by 12 planned post hoc *t*-tests with Holm-Sidak correction to reduce the risk of type 1 error associated with multiple comparisons. *P < 0.05; **P < 0.01.

Renal gene expression of the angiotensin receptors and ACE2 were expressed relative to the vehicle-treated adult WT mice. There were no differences in AT_1b_R, ACE2, or MasR gene expression between the vehicle-treated groups (Figure 2B,D,E). In vehicle-treated mice, renal expression of the gene for the AT_1a_R was similar between adult WT and AT_2_R-KO mice (Figure 2A). In both genotypes, expression of the gene for the AT_1a_R was 2.5-fold greater in aged versus adult mice (both *P* < 0.001, Figure 2). In contrast, expression of the gene for the AT_1a_R was 50% lower in aged-OVX WT than aged WT mice (*P* = 0.02, Figure 2A). Renal expression of the gene for the AT_2_R was 52% lower in aged WT mice as compared to adult WT mice (*P* = 0.03, Figure 2C).

Following a 14-day AngII treatment, renal AT_1a_R expression was ~3-fold higher in adult WT (*P* = 0.002) and ~2.5-fold higher in adult AT_2_R-KO (*P* = 0.02) female mice than their vehicle-treated counterparts (Figure 2A). AT_2_R expression was ~1-fold higher in AngII than vehicle-treated aged WT mice (*P* = 0.003, Figure 2C). In comparison, there were no differences in renal AT_2_R expression between vehicle and AngII treated adult WT and aged-OVX WT mice (Figure 2C). In aged WT mice, ACE2 gene expression was 2-fold higher in AngII than vehicle-treated mice (*P* = 0.009, Figure 2D). In response to AngII, ACE2 gene expression was ~45% lower in adult and aged AT_2_R-KO mice as compared to WT counterparts (Figure 2D). In aged WT mice, ACE2 gene expression was 70% lower with OVX (*P* = 0.03, Figure 2D). There were no significant differences in AT_1b_R or MasR gene expression in response to AngII in any group (Figure 2B,E).

## 4 Discussion

The main findings of the present study were that (i) the AT_2_R attenuates pressor responsiveness to AngII in adult female mice of reproductive age, in agreement with our previous finding [4], (ii) with age, WT female mice have an enhanced pressor response to AngII which is similar to that observed in adult AT_2_R-KO mice, (iii) pressor responsiveness to AngII is not increased further with age in AT_2_R-KO female mice, and (iv) aging in WT mice is associated with a shift in the depressor/pressor balance of the renal RAS. Thus, the enhanced pressor responsiveness to AngII observed with age in WT female mice might be, at least in part, due to a shift in the depressor/pressor balance of the renal RAS and/or deficits in the downstream AT_2_R signaling pathways.

There was no difference in basal MAP between adult WT and AT_2_R-KO female mice. Moreover, we did not detect an increase in arterial pressure with age. This latter finding is consistent with our recent work which also observed no significant difference in basal arterial pressure between 4-month-old and 14-month-old female mice [15]. Similar to the increase in arterial pressure observed in postmenopausal women, which occurs in the 10–15 years following menopause, we propose that a time lag exists between reproductive senescence and the increase in arterial pressure in aged female rodents. Reproductively senescent spontaneously hypertensive rats (SHR) have many of the hallmarks associated with the increase in arterial pressure observed in postmenopausal women, including increased plasma renin activity [16]. However, it has been demonstrated that although reproductively senescent SHR cease regular estrus cycling at 10 months of age, the rise in arterial pressure to match that measured in males is apparent by 18 months of age [16],[17]. Likewise, we have recently observed a similar phenomenon in aged mice. Consistent with previously published studies, which have demonstrated that mice cease regular estrus cycling by 8–14 months of age [18],[19], in the present study and our earlier work, our cohorts of aged female mice enter reproductive senescence by 14 months of age [15]. However, the increase in arterial pressure in reproductively senescent female mice is apparent at 18 months of age, at which time the sexual dimorphism in arterial pressure is lost in mice (arterial pressure measured via radiotelemetry, unpublished data).

In the present study, AT_2_R deficiency augmented pressor responsiveness to AngII in adult female mice. This finding is consistent with our earlier work, demonstrating that the AT_2_R plays a protective role in the regulation of arterial pressure in adult female rodents [4],[8]. In these studies, AT_2_R deficiency in adult mice and pharmacological blockade of the AT_2_R in adult rats augmented the pressor response to AngII in females [4],[8]. Moreover, we showed that in response to a low dose of AngII, arterial pressure was paradoxically decreased in adult female rats, an effect that was inhibited by AT_2_R blockade [8]. Given that females have a greater AT_2_R to AT_1_R balance than males [5],[15],[20],[21], that the expression of depressor components (ACE2/Ang(1-7)/MasR axis and the AT_2_R) of the RAS is enhanced in females [7], and that AngII has ~15-fold greater affinity for the AT_2_R than the AT_1_R [22], these suggest that similar mechanisms underpin the depressor response to a low-dose AngII and the attenuated pressor response to a high-dose AngII infusion observed in female rodents. Thus, in response to an AngII infusion in female rodents, AngII will bind with greater affinity for the AT_2_R than the AT_1_R. Moreover, AngII is also converted to AngIII and Ang(1-7), both of which activate the AT_2_R to stimulate arterial pressure lowering effects [23]-[25]. Indeed, in the present study we observed an increase in renal ACE2 mRNA in AngII-treated adult and aged WT mice treated.

While there is speculation in the literature that the protective effects of estrogen on the regulation of arterial pressure in females may be lost with age, this assumption is based upon the evidence obtained from adult OVX animals. For example, it has been demonstrated that the pressor response to AngII in adult mice is augmented by OVX and restored by estrogen replacement [9],[26]. Similarly, we have previously shown that in adult rats the arterial depressor response to a low-dose AngII infusion is abolished by OVX and restored by estrogen replacement [13]. While these animal models are useful for understanding the contribution of estrogen to the normal regulation of arterial pressure in adult females, they do not accurately represent the cardiovascular and renal adaptations that accompany the aging process [27]. As a result, attempts to mimic menopause and loss of estrogen in adult OVX animals do not take into account the effects of aging, including increased RAS activation, which may influence angiotensin receptor levels independent of alterations in circulating estrogen levels [16],[28],[29].

In the present study performed in aged females, we clearly demonstrate an increase in the pressor responsiveness to AngII in aged WT female mice. Moreover, the enhanced pressor response to AngII in aged WT female mice was of a similar magnitude to that observed in adult AT_2_R-KO female mice. In contrast, in AT_2_R-KO female mice, there was no difference in the pressor response to AngII with age. Together these findings suggest that the increase in pressor responsiveness to AngII is due to AT_2_R deficiency. At present, it is unclear if this AT_2_R deficiency is due to an age-related reduction in AT_2_R expression and/or activation. For example, in vehicle-treated mice, the renal AT_2_R/AT_1a_R mRNA expression balance was lower in aged than adult WT female mice. Thus, normal aging decreases renal AT_2_R mRNA expression in reproductively senescent female mice. However, during pathological situations, such as AngII-induced hypertension, there is a compensatory increase in renal AT_2_R mRNA expression in aged reproductively senescent female mice. Despite the increase in renal AT_2_R mRNA expression, the protective effects of AT_2_R activation were not observed in reproductively senescent female mice. This latter finding suggests that the renal AT_2_R signaling pathways may be impaired with age. Consistent with this hypothesis, renal ACE2 mRNA expression was similar in AngII-treated adult and aged WT mice. This may suggest that there is a similar level of stimulation of the AT_2_R by ACE2-generated Ang(1-7) in both adult and aged reproductively senescent female mice during AngII-induced hypertension.

However, it is important to note that the current study measured mRNA gene expression, which does not necessarily reflect protein expression or activity. Moreover, mRNA gene expression was determined in transverse kidney sections, which may mask subtle differences in discrete regions of the kidney. Therefore, further investigation of how the protective effects of renal AT_2_R activation are lost with age is warranted.

We have gained strong evidence that the AT_2_R elicits sex-specific effects on arterial pressure and renal function in adult rodents. Within the kidney, the AT_2_R maintains autoregulation of renal blood flow and glomerular filtration rate (GFR) at low renal perfusion pressures in females and provides protection against the vasoconstrictor effects of AngII [30]. The sensitivity of the tubuloglomerular feedback mechanism, which is a major regulator of renal vascular tone and thus GFR, to AngII is reduced by the presence of the AT_2_R in female but not male mice [4]. It is well documented that the AT_2_R elicits its effects by activating the nitric oxide (NO)-cyclic GMP (cGMP) pathway. In aging (8-month-old) SHR, it has been demonstrated that estrogen-dependent activation of the NO-cGMP pathway is reduced [31]. Given that estrogen increases AT_2_R expression, loss of estrogen-dependent activation of the NO-cGMP pathway may in part be due to a reduction in AT_2_R function.

In the present study, we were unable to reliably measure plasma estrogen levels as murine plasma estrogen levels were below the detection limits of commercially available estrogen assays. This is consistent with previously published studies, which have demonstrated that commercial estrogen assays lack sensitivity and specificity in female mice [32],[33]. However, we did assess vaginal cytology and confirmed that our cohort of aged mice had entered reproductive senescence. Moreover, we included an aged-OVX cohort in the present study to account for residual effects of ovarian-produced estrogen. While we did not detect differences between aged and aged-OVX female mice with respect to basal MAP or pressor responsiveness to AngII, subtle differences were observed in renal gene expression of RAS components, indicating that an aged-OVX mouse is not equivalent to an aged (ovary-intact) mouse. Our conclusion is also supported by previous studies that have observed differential effects of adult-OVX, aged-OVX, and aging alone, demonstrating that an OVX female, be it adult or aged, is not equivalent to an aged ovary-intact female [16],[34]. Since the ovaries produce sex hormones other than estrogen, including testosterone, progesterone, and relaxin, alterations in the sex hormone milieu of aged females, as observed in reproductively senescent SHR [16], may play a pivotal role in the loss of the estrogen-dependent activation of NO-cGMP pathway. This may be of particular importance given that recent evidence indicates that heterodimer complexes formed between the relaxin family peptide receptor 1 and the AT_2_R activate the NO-cGMP pathway [35]. Certainly, it has been demonstrated that the AT_2_R contributes to the ability of relaxin to mediate renal anti-fibrotic effects *in vivo*[35]. Hence, both estrogen and relaxin may be required to elicit the arterial pressure lowering effects of the AT_2_R.

To the best of our knowledge, the activation of the protective RAS depressor pathways is yet to be measured in postmenopausal women. However, it is tempting to speculate that the balance of the RAS is shifted postmenopause towards the pressor pathway. Certainly, it is known that plasma renin activity is increased in postmenopausal women compared to premenopausal women [36],[37]. Moreover, AT_1_R antagonism improves vascular function in postmenopausal women who are not receiving hormone replacement therapy [38]. Additionally, treatment with candesartan unmasks a vasodilator response to AngII in forearm resistance vessels of elderly women (67 years), an effect that is partially blocked by co-infusion of the AT_2_R antagonist PD123319 [39]. Consistent with a decrease in the depressor/pressor balance of the RAS in aged females, increased AT_1_R activation may potentiate other vasoconstrictive mechanisms including endothelin-1 and reactive oxygen species, which are thought to contribute to the age-related increase in arterial pressure in postmenopausal women [17].

## 5 Conclusions

Understanding the mechanisms contributing to the increase in arterial pressure in females with aging is of the utmost importance. Our study in mice demonstrates that pressor responsiveness to AngII is increased following reproductive senescence, and moreover, that this is associated with loss of the protective depressor RAS pathways. These findings provide novel insight into age-related changes in the RAS and imply that different therapeutic strategies may be required to effectively treat hypertension in premenopausal and postmenopausal women.

## Competing interests

The authors declare that they have no competing interests.

## Authors’ contributions

KMM helped designed the study, performed all the experiments and statistical analyses, and drafted the manuscript. LMH participated in the gene expression experiments and helped draft the manuscript. GAH participated in the interpretation of the data and revision of the manuscript. REW participated in the design of the study, interpretation of the data, and revision of the manuscript. KMD conceived and designed the study and helped draft the manuscript. All authors read and approved the final manuscript.

## Authors’ information

This work was supported by the National Health and Medical Research Council of Australia (project grants #490919 and #606652) and the National Heart Foundation of Australia (project grant G11M5816). KMD was supported by a National Health and Medical Research Council of Australia research fellowship #1041844.
